# Lectures on the Theory and Practice of Surgery

**Published:** 1831

**Authors:** 


ART. VIII.—*Lectures on the Theory and Practice of Surgery.*—By JOHN ABERNETHY, F. R. S. &c. C. S. Francis, N. York: 1830. pp. 190.

In a former number of this Journal, a very full review of the surreptitious lectures of Mr. Abernethy were given. The publication of these lectures gave great annoyance to the author, from the impression that it was a gross invasion of his rights, and a serious injury to his reputation. Independent of the disadvantage to which every extemporaneous lecturer must appear, when his unguarded remarks, transcribed by a careless and perhaps not very competent stenographer, are subjected to the criticism which we are ready to apply to the works of so distinguished an author, those who have read the work to which we refer, will recollect that Mr. A. was constantly in the habit of divesting himself of his professional dignity, and of placing himself before his students in an attitude which would add little to his reputation, in the estimation of those, whose approbation he would justly esteem the true criterion of merit.

It is, therefore, a matter of no surprise, that a sense of the injury inflicted by this unauthorized publication, should induce the author to use the following language, upon this occasion: "When the lectures of certain teachers, as of Boerhaave, Cullen, and Black, had acquired a high degree of celebrity, they were indeed surreptitiously published by persons who were naturally ashamed to acknowledge their acts; it has, therefore, been left to the present era to produce a character, in the editor of a periodical publication called the Lancet, so devoid of all good feeling and all sense of shame, as to avow and defend conduct so unprincipled."

The lectures were originally published in the Lancet, and Mr. A. having ineffectually attempted to arrest their publication by a legal process, and they being again reprinted both in Great Britain and this country, he conceived it necessary, in self defence, to offer to the world the book now before us.

The design of this work is limited to the doctrinal part of surgery, and to those principles of practice from which Mr. A. thinks, the principal interest of his lectures arose. It will contain, therefore, a very imperfect account of the lectures delivered by the author, since he has omitted almost all reference to anatomy and physiology. He has also, for the sake of brevity, avoided repeating any thing that may be found either in his works, or in those elementary treatises that are in the library of every practitioner. It is, in a word, designed to be simply a record of his own experience and observation; the remarks of others being only introduced, so far as may be necessary to enable him to present his own views in a regular system.

The example of Mr. Abernethy, in this respect, is worthy of all imitation, and may advantageously be put in comparison with that of some American physicians who have taken advantage of their popularity, repeatedly to sell new copy rights of the same ideas, differently modelled, or presented under a new arrangement; and, what adds to the imposition, adapted to the same class of readers. Such authors, to use the idea of Dr. James Johnson, exhibit their learning by showing their familiarity, not with the writings of other authors, but with their own.

It has been remarked that it is impossible for a poet or a painter to produce more than one production perfectly original; the characters of every succeeding piece being modelled after the standard which the author had in view in the production of his maiden effort. To a certain extent, this remark applies to Mr. Abernethy's writings. In every part of them, we see the same principles extended to the various forms of disease, that come under his observation. Irritation and derangement of the digestive organs, are, with him, the box of Pandora. To urge the importance of soothing the one, and of correcting the other, is the great object of all his writings; and in every page, he illustrates the importance of these principles, by extending their application to some new form of disease. He every where manifests that peculiar tact, that mechanical genius, which is indispensable to the great surgeon, and in the improvements which his inventive genius has introduced into the practice of his profession, he has not been surpassed by any of his contemporaries.

The copious extracts which have been made from his surreptitious lectures, will serve us the necessity of making many from the present work. His remarks upon the treatment of oblique fractures of the thigh, will be read with interest.

"Having been instructed in the early part of my life to put up fractures of the lower extremity in what was called a relaxed position of the limb, I adhere to this practice from a conviction of its excellence. In the cases however, of oblique fractures of the leg in which the lower portion of the bone is retracted, and which require support from behind, it seems better that the limb should be laid over the double inclined plane, as has been already observed. To accomplish the object of keeping the lower extremity invariably under the circumstances described, namely, every longitudinal inch of the limb resting upon the plane opposite it—it is requisite that the trunk of the patient should be fixed on its side. To effect this, it is necessary that each anterior superior spine of the ilium and the acromion of either scapula should be in the same perpendicular plane. When we lie down to sleep, and turn completely on our sides, in this position we wake without the slightest change. A person laid in this position for a certain time, makes a kind of cast in the bed in which he lies; and this subsequently supports him in the proper posture. A drawn sheet is to be put under the patient, and a sheet of strong pasteboard covered with a napkin may be slid under the buttocks, when the fæces are to be discharged, by which means they may be removed without soiling the bed-clothes. I have seen patients lie for three months in this position without material inconvenience. I have known individuals who have twice had their thighs broken, and who, treated the one time by being laid on the back with the limb resting on the double inclined plane, the other by being placed on the side, have given a decided preference to the latter position, although it happened that the union of the fracture was much more tardy on this than on the former occasion. The lateral position of the limb seems to me to be particularly advantageous in fractures of the neck of the thigh bone, where the base of the arch is separated from the shaft of the bone, as it keeps the broken bone steadily pressed against the part from which it has been detached. I have heard this position objected to, on account of its rendering it difficult to compare the length of the limbs with one another; which, however, may be done with as much accuracy as when a patient is laid upon his back. In the latter case we can never be assured that the limbs are equal, unless the two anterior superior spines of the ilium are in a horizontal line. If the two patellæ are then in a parallel line, it is evident that the limbs are of equal length. If a person lie on his back, with the anterior superior spines of the ilium in the same horizontal line, having the lower extremities extended and the foot straight forward, then will a line, drawn at a right angle with the former, from the centre of the patella, pass over the middle of the ankle joint, the dorsum of the foot, and that toe next the great toe. Under these circumstances, there can be neither abduction nor adduction, eversion nor inversion, of the thigh, and we are assured that the limb is straight. This observation can be made with equal certainty when a person lies upon his side; for if the two anterior superior spines of the ilium be in the same perpendicular line, and the patellæ correspond, we have an assurance that the limbs are of equal length. When a person has broken the neck of the thigh bone, and retraction has taken place, we may, by lightly rubbing the limb, and using gentle extension of the knee, gradually bring the thigh to its full length; yet it is apt to be retracted again, when these measures are discontinued, by the action of the irritable muscles. Permanent extension, in a degree adequate to counteract the action of the muscles, may in this case also be applied. A soft handkerchief may be wrapped round the knee, to which a cord may be attached and trained over a pulley screwed into the edge of the bedstead. A light weight, yet adequate to prevent such retraction, may be appended to this cord. In these fractures, as in others, the irritable state of the muscles is but of short duration; and when the bones have rested for a short time in their proper situation and become agglutinated, the muscles will rather tend to maintain them in their proper position than to alter it."

The following remarks upon the reduction of dislocations, are so very important, and display so much of the peculiar surgical genius, which does this great man so much honor, that we cannot resist the temptation to introduce them.

"As the ligaments and capsules of joints are inelastic, so are they in cases of dislocation always sufficiently lacerated to admit of the bones being replaced in their proper situations, were not the attempt to do this opposed by the muscles. But the injury has put these active organs into a state of apparent alarm, and they most vigilantly and powerfully resist every attempt at motion of the dislocated bone, as if fearful of farther mischief being done. We are, therefore, under the necessity either of eluding their vigilance, or of overcoming their power. The former cannot in general be expected to succeed, although it may occasionally be accomplished. Should a surgeon be inclined to make the attempt, he proceeds in the following manner:—We shall suppose the case to be that in which the head of the os brachii is dislocated in the axilla, and rests just below the glenoid cavity of the scapula. Having seated the patient, bent the elbow, and turned the hand supine, the surgeon requests him to support the weight of the fore-arm with the other hand, telling him that he wishes to be assured of the exact nature of the accident before he proceeds to do any thing, and that the examination will be attended with no pain. The surgeon then grasps the elbow, applying his fingers and thumb to the opposite condyles of the os brachii, so as to command the motions of the bone, which he then moves gently forwards and backwards, and in a direction from and towards the patient's body. In attempting these motions he finds himself resisted by the muscles, and apprises the patient that this action deprives him of the power of examining, and that if the arm were but left perfectly at liberty, the exact nature of the accident would be speedily and satisfactorily ascertained. He inquires whether the motions he makes are productive of any pain; and when the patient finds that they are not, he will allow the surgeon to move the arm more freely than before. The surgeon now engages the patient upon some topic calculated to fix his attention by the interest it excites, for example, on the manner in which the accident occurred, and then suddenly, and when the patient is off his guard, moves the head of the bone in that direction which tends to replace it in its socket. An attempt to elude the vigilance of the muscles is more likely to succeed after previous unsuccessful efforts to reduce the dislocation have been made; for the patient is then fearless of the trivial motions which the surgeon feels obliged to practice, and the muscles are also weary and disposed to rest. I have known several instances in the course of my life of dislocations being reduced in this manner, both before and after the ordinary methods had been tried without success."

Having failed to reduce the dislocation by art or force, the author recommends to weary out the muscles by slight but continued extension, kept up for a considerable time.

"Passing through the square of St. Bartholomew's Hospital one summer afternoon, when the weather was very warm, I observed a number of students standing at one of the doors wiping the perspiration from their faces, aud panting from heat and exertion. They told me they had been trying for an hour to reduce a dislocated shoulder without avail, for the strength of the patient was immense. The man was a coal heaver, and his muscles were so bulky that they appeared to me to equal those of the Farnese Hercules; nevertheless he appeared to be nearly as much exhausted as his medical attendants.—Being unwilling to lose what appeared a very favourable opportunity for attempting reduction, I asked the patient if he would allow me, upon the following conditions, to try to replace the bone:—1st, That I should give him no pain; and, 2d, That the attempt should not be continued longer than ten minutes. He consented to my proposition with a smile. Having fixed the trunk of the body and lower costa of the scapula by means of a sheet, I applied a napkin above the condyles of the os brachii; and, having twisted it so as to fix it completely, gave the ends to two of the heaviest of the students present, telling them to be sure to prevent the napkin from becoming untwisted and slipping over the elbow joint; and having brought the ends of it close to their own bodies, to lean their weights upon them; for this force I knew they could continue unremittingly without fatigue. This being done, I inquired of the patient whether he was in any pain, the first condition being that he should suffer none. He having answered in the negative, I looked at my watch and said, "Now, sir, ten minutes are all I shall require;" and he smiled in derision, saying it would never answer. But before five minutes had elapsed he expressed himself in a very impatient tone, earnestly desiring that the effort, which he was sure would be unavailing, might be discontinued. I reminded him of our compact, showed him my watch, and told him the time would soon elapse. In two minutes more he declared very angrily that he would not endure it any longer, said he should faint, and at that moment the bone slipped into its socket."

We are not certain that this work will fulfill the design of the author, by superceding the one which gave him so much uneasiness; nor is it perhaps very desirable that it should. The present volume will be the companion of men of science; the other possesses attractions to the student, that will always make it popular to that class of readers. Nor are we disposed to judge very censoriously, the burlesque mode of lecturing, which Mr. A. adopted with so much effect.

The most important, we would say almost the sole object of lecturing, is, to fix the attention of the pupil upon facts, which, in reading, he passes over with little interest; and although we would recommend to every professor to cultivate the higher arts of eloquence, yet these are so seldom met with, that gratitude is due even to the speaker who accomplishes the same object, by peculiarity of manner, or some humbler qualification.

